# Unique regulation of Na‐glutamine cotransporter SN2/SNAT5 in rabbit intestinal crypt cells during chronic enteritis

**DOI:** 10.1111/jcmm.13257

**Published:** 2017-12-22

**Authors:** Soudamani Singh, Subha Arthur, Uma Sundaram

**Affiliations:** ^1^ Department of Clinical and Translational Sciences Appalachian Clinical and Translational Science Institute Joan C. Edwards School of Medicine Marshall University Huntington WV USA

**Keywords:** Glutamine transport, SN2/SNAT5, leukotrienes, prostaglandins and crypt cells

## Abstract

The only Na‐nutrient cotransporter described in mammalian small intestinal crypt cells is SN2/SNAT5, which facilitates glutamine uptake. In a rabbit model of chronic intestinal inflammation, SN2 stimulation is secondary to an increase in affinity of the cotransporter for glutamine. However, the immune regulation of SN2 in the crypt cells during chronic intestinal inflammation is unknown. We sought to determine the mechanism of regulation of Na‐nutrient cotransporter SN2 by arachidonic acid metabolites in crypt cells. The small intestines of New Zealand white male rabbits were inflamed *via* inoculation with *Eimeria magna* oocytes. After 2‐week incubation, control and inflamed rabbits were subjected to intramuscular injections of arachidonyl trifluoromethyl ketone (ATK), piroxicam and MK886 for 48 hrs. After injections, the rabbits were euthanized and crypt cells from small intestines were harvested and used. Results: Treatment of rabbits with ATK prevented the release of AA and reversed stimulation of SN2. Inhibition of cyclooxygenase (COX) with piroxicam did not affect stimulation of SN2. However, inhibition of lipoxygenase (LOX) with MK886, thus reducing leukotriene formation during chronic enteritis, reversed the stimulation of SN2. Kinetic studies showed that the mechanism of restoration of SN2 by ATK or MK886 was secondary to the restoration of the affinity of the cotransporter for glutamine. For all treatment conditions, Western blot analysis revealed no change in SN2 protein levels. COX inhibition proved ineffective at reversing the stimulation of SN2. Thus, this study provides evidence that SN2 stimulation in crypt cells is mediated by the leukotriene pathway during chronic intestinal inflammation.

## Introduction

The key nutrient of the mammalian small intestinal enterocyte is the amino acid glutamine 1. This conditionally essential amino acid is absorbed by different Na‐glutamine cotransporter (NGcT) processes in the mammalian small intestinal villus and crypt cells 2. The predominantly absorptive villus cells assimilate glutamine *via* B0AT1 (SLC6A19) while the crypt cells absorb it *via* SN2/SNAT5 (SLC38A5) 2, 3. In fact, this is the only nutrient absorptive process that has been conclusively demonstrated in the small intestinal crypt cells to date 4, 5, 6, 7, 8, 9, 10. In both cell types, this secondary active process is dependent on the basolateral membrane (BLM) Na^+^/K^+^‐ATPase to provide the favourable transcellular sodium gradient 2.

Glutamine has also been shown to play an important role in maintaining mucosal health and integrity 11, 12. The mature epithelial mucosal cells lining the intestinal tract of many animals are replaced completely every 2–3 days 13. The loss of these cells, which occurs at the villus tips, is balanced by continuous replication of cells, a process that is restricted to the lower two‐thirds of the crypt region 14. The constant renewal of the epithelium of this highly metabolically active tissue is a process that requires a ready supply of glutamine 13. Glutamine is not only necessary as a nutrient for intestinal proliferation and renewal 1, 13, 15, but also important for mediating the proliferation by growth factors 1, 15, 16. Glutamine also plays an essential role in the regulation of cell‐specific processes including metabolism (*e.g*. oxidative fuel, gluconeogenic and lipogenic precursor), cell integrity (apoptosis, cell proliferation), protein synthesis and degradation, contractile protein mass, redox potential, extracellular matrix synthesis and gut barrier functions 15, 16.

Glutamine may be even more important in intestinal pathophysiological states affecting the intestinal mucosa 17. Inflammatory bowel disease (IBD) is characterized by chronic mucosal inflammation of the gastrointestinal tract 18. A significant consequence of mucosal inflammation is malabsorption of electrolytes, water and nutrients. Intestinal malabsorption in IBD may be due to alterations in motility, the enteric nervous system and blood flow. At the mucosal level, malabsorption may result from atrophy of the absorptive villus cells and hyperplasia of the secretory crypt cells. Most, if not all, alterations in absorptive processes are thought to be caused by immune‐inflammatory mediators released by immunocytes during chronic intestinal inflammation. However, how the altered immune system in IBD may contribute to the pathogenesis of nutrient malabsorption is poorly understood 18, 19, 20.

Studies have shown that arachidonic acid (AA) metabolites (AAM) of the COX and LOX pathways are known to be increased in IBD mucosa 21. AA is a key component of the plasma membrane. Various physiological and pathological stimuli can result in the activation of phospholipase A2 (PLA2) to liberate AA from membrane phospholipids into the cytoplasm 22. Upon release, AA is converted into unstable endoperoxide intermediates, like prostaglandins (PGs) 22, 23 by the action of COX and leukotrienes (LTs) by the action of LOX 22. PGs and LTs have been implicated in the pathogenesis of a number of diseases including rheumatoid arthritis, asthma, psoriasis, multiple sclerosis and IBD. For instance, in ileal and colonic mucosa of patients with IBD, there is an increase in the types and amount of PGs and LTs 22, 23, 24. These observations suggest regulation of AA release and/or AAM synthesis may be areas of therapeutic strategy for IBD.

Presently, it is not known whether a given immune‐inflammatory mediator/pathway is responsible for alterations seen with a specific glutamine absorbing transport pathway during chronic enteritis. Glucocorticoids are a common treatment for IBD, and as nonspecific immune modulators, they are known to block multiple pathways of the immune system including the AAM cascade. Indeed, the glucocorticoid methylprednisolone has been shown to alleviate the stimulation of glutamine transport *via* SN2 in the rabbit small intestine 25. However, long‐term treatment with glucocorticoids is fraught with innumerable significant side effects. Therefore, it is necessary to identify the specific immune‐inflammatory mediator/pathways that might be responsible for the alteration of glutamine absorption during chronic enteritis. However, there are no studies on whether AA, AAM, PGs or LTs may regulate NGcT during chronic intestinal inflammation. Thus, the aim of this study was to uncover whether AAM may be the cause of the alteration of NGcT SN2 activity in crypt cells observed during chronic enteritis.

## Materials and methods

### Animals

New Zealand white male rabbits weighing between 2.0 and 2.2 kg were procured from Charles River Laboratories (Spencerville, OH, USA). All the rabbits were housed in an individual stainless steel cages in an animal facility maintained at 22 ± 2°C with humidity 50–70% and 12‐hrs light and dark cycles. All the rabbits had free access to rabbit diet (5321‐Laboratory Rabbit Diet; PMI nutrition International, St. Louis, MO, USA) and water. The rabbits were acclimatized for 1 week before the study. All of the procedures like handling, treatments and euthanization were carried out in accordance with a protocol approved by the Institutional Animal Care and Use Committee of Marshall University.

### Initiation of chronic inflammation

Eimeria magna, a natural parasite of rabbits, was used to induce intestinal inflammation in the experimental rabbits. Chronic inflammation thus induced in rabbits causes symptoms quite similar to IBD diagnosed in humans 4. Briefly, the rabbits were intragastrically inoculated with approximately 20,000 coccidia in saline. The animals develop chronic intestinal inflammation with the peak being on day 14. The animals were sacrificed on day 14. Control animals were inoculated with just saline.

### Drug treatment

After 1 week of acclimatization, all the animals were divided into eight groups, each group containing four animals. Group 1 contained control rabbits which were treated with an intramuscular (I.M.) injection of saline. In group 2, chronic intestinal inflammation was induced in rabbits as described above and previously reported 5. Group 3 contained normal rabbits treated with 3 mg/kg body weight I.M. injection of ATK. Animals in group 4 were inflamed rabbits treated with 3 mg/kg body weight of I.M. injection of ATK (days 12 and 13 post‐inoculation). Group 5 had normal rabbits treated with I.M. injection of 10 mg/kg body weight piroxicam (PRX). Group 6 contained inflamed rabbits treated with I.M. injection of 10 mg/kg body weight PRX (days 12 and 13 post‐inoculation). Group 7 had normal rabbits treated with I.M. injection of 0.5 mg/kg body weight MK886. Group 8 contained inflamed rabbits treated with I.M. injection of 0.5 mg/kg body weight MK886 (days 12 and 13 post‐inoculation). At the end of the experimental period, all the inflamed rabbits and inflamed drug‐treated rabbits were sacrificed on day 14 post‐inoculation.

### Cell isolation

Small intestinal crypt cells were isolated by calcium chelation technique as previously described 5. Briefly, a 3‐ft section of ileum was filled and incubated with cell isolation buffer (0.15 mM EDTA, 112 mM NaCl, 25 mM NaHCO3, 2.4 mM K2HPO4, 0.4 mM KH2PO4, 2.5 mM L‐glutamine, 0.5 mM β‐hydroxybutyrate and 0.5 mM dithiothreitol; gassed with 95% O2, and 5% CO2, pH 7.4, at 37°C) for 3 min. and was gently palpitated for another 3 min. to facilitate cell dispersion. The buffer was then drained out from the ileal section, phenylmethylsulfonyl fluoride was added, and the suspension was centrifuged at 100 *g* for 3 min. Enzyme markers, morphology and transporter specificity were used to confirm the purity of isolated crypt cells. Isolated crypt cells were flash frozen immediately in liquid nitrogen and stored at −80°C until further required for brush border membrane vesicle (BBMV) preparation.

### Na^+^/K^+^‐ATPase measurement

From cellular homogenates, Na^+^/K^+^‐ATPase activity was measured as Pi liberated from the same amount of cells from normal or inflamed intestine as previously described 26. Na^+^/K^+^‐ATPase enzyme‐specific activity was expressed as nanomoles of Pi released per milligram protein per minute.

### Uptake studies in crypt cells

Na‐glutamine uptake in intact crypt cells was done using a previously described protocol 2, 27. Crypt cells (100 mg wet wt.) were washed and resuspended in HEPES buffer containing 0.2 mM glutamine, 4.5 mM KCl, 1.2 mM KH2PO4, 1.0 mM MgSO4, 1.25 mM CaCl2, 20 mM HEPES, and either 130 mM sodium chloride or choline chloride and was gassed with 100% O2 (pH 7.4 at 37°C). Ten μCi of ^3^H‐glutamine was added to 1 ml of cell suspension in HEPES buffer, and 100 μl aliquots were removed at 2 min. and mixed with 1 ml of ice‐cold stop solution (choline‐HEPES buffer) to stop the uptake. The mixture was then filtered on 0.65‐μm Millipore (Bedford, MA, USA; HAWP) filters and washed twice with ice‐cold stop solution. The filter was dissolved in 5 ml of Ecoscint solution, and the radioactivity was determined in a Beckman Coulter LS6500 Scintillation counter.

### BBMV preparation and uptake studies

Crypt BBMVs were prepared by CaCl_2_ precipitation and differential centrifugation as previously described 2, 27. A rapid filtration technique was employed to do the uptake studies as described earlier 2, 27. In brief, 5 μl of BBMV resuspended in vesicle medium (100 mM choline chloride, 0.10 mM MgSO4, 50 mM HEPES–Tris (pH 7.5), 50 mM mannitol, 50 mM KCl) was voltage clamped with 10 μM valinomycin and 100 mM carbonyl cyanide p‐(tri‐fluoromethoxy) phenyl‐hydrazone (FCCP). The vesicles were then incubated in 95 μl of reaction medium (50 mM HEPES–Tris buffer (pH 7.5), 0.2 mM glutamine, 20 μCi ^3^H‐glutamine, 0.10 mM MgSO4, 50 mM KCl, 50 mM mannitol, and 100 mM of either NaCl or choline chloride), and at desired time‐points, the uptake was arrested by mixing the solution with ice‐cold stop solution made of 50 mM HEPES–Tris buffer (pH 7.5), 0.10 mM MgSO4, 75 mM KCl and 100 mM choline chloride. A 0.45‐μm Millipore (HAWP) filter was used to filter the stopped reaction mixture, and the filter was washed twice with 10 ml of ice‐cold stop solution. Ecoscint solution (5 ml) was used to dissolve these filters, and radioactivity was determined in a Beckman Coulter LS6500 Scintillation counter.

### Western Blot analysis

Western blot experiments were performed with a custom‐made antibody raised in chicken against rabbit SN2/SNAT5. The use of this antibody has been well validated through our previous publications 2. BBM protein was prepared from all the groups for Western blot experiments. The BBM was solubilized in a RIPA lysis buffer (sc‐24948, Santa Cruz Biotechnology Inc., Santa Cruz, CA, USA). Protein concentrations were determined using a NanoDrop 2000 Spectrophotometer (Thermos Scientific, Wilmington, DE, USA). Proteins were separated by electrophoresis on a 10% gradient gel (Bio‐Rad Laboratories, Hercules, CA, USA). Proteins on the gel were transferred to a polyvinylidene membrane which was blocked with 5% bovine serum albumin in tris buffered saline with tween 20 (T‐9039, Sigma‐Aldrich, St. Louis, MO, USA). The blot was incubated with the primary antibody and incubated overnight at 4°C followed by incubation with an antichicken IgY antibody conjugated to horseradish peroxidase (Jackson Immuno‐Research Laboratories, West Grove, PA, USA) for an hour at room temperature. Enhanced chemiluminescent (ECL) reagent for Western blotting detection (RapidStep™ ECL Reagent; Millipore) was used to detect the immobilized protein. The resultant chemiluminescence was detected using biomax film (Kodak, Rochester, NY, USA), and the intensity of the bands was analysed by FluorChem™ instrument (Alpha Innotech, San Leandro, CA, USA).

### Statistical analysis

Results are shown as mean ± standard error of mean (S.E.M.) in all figures calculated with GraphPad Prism 7 (San Diego, CA, USA) program. All individual uptakes were performed in triplicate. The ‘*n*’ number for any set of uptake experiments or Western blot analysis refers to vesicle or isolated cell preparations from different animals. Data were analysed using one‐way analysis of variance (anova) to assess the significance between control and experimental groups using GraphPad Prism 7 for statistical analysis. A *P* value of less than 0.01 was considered significant.

## Results

### Effect of ATK on NGcT in the intact crypt cells

**Figure 1:**
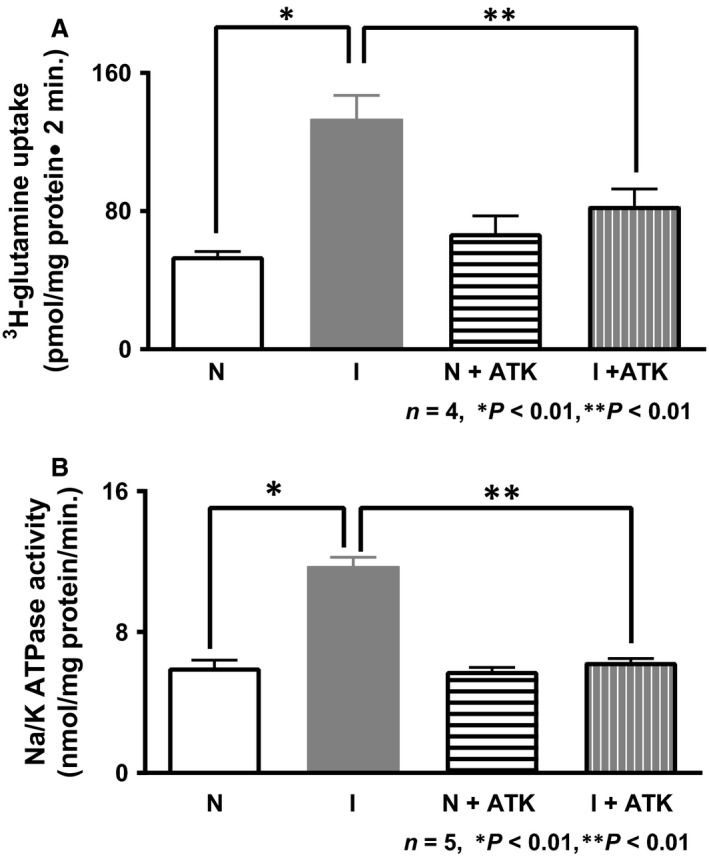
(**A**) Effect of ATK on SN2 in intact crypt cells. Na‐dependent glutamine uptake was significantly increased in crypt cells from the chronically inflamed intestine. Rabbits with chronic enteritis treated with ATK reversed NGcT stimulation. ATK had no effect on NGcT in crypt cells from the normal intestine. (**B**) Effect of ATK on Na^+^/K^+^‐ATPase. Na^+^/K^+^‐ATPase activity was significantly increased in the crypt cells during chronic enteritis and was reversed by ATK treatment. Na^+^/K^+^‐ATPase activity in ATK‐treated normal intestinal crypt cells remained unaffected.

NGcT was significantly stimulated in intact crypt cells from the chronically inflamed intestine. ATK treatment abolished this stimulation. ATK did not have any effect in the crypt cells from the normal intestine (Fig. 1A; 53.9 ± 2.7 pmol/mg protein 2 min. in normal; 133.5 ± 13.5 in inflamed; 67.3 ± 10 in normal+ATK; 83 ± 9.8 in inflamed+ATK; *n* = 3, *P* < 0.01). These data indicated that the metabolites of AA pathway likely mediate the regulation of NGcT in crypt cells from the chronically inflamed intestine.

### Effect of ATK on Na^+^/K^+^‐ATPase activity in crypt cells

Na‐dependent cotransporters require the Na^+^/K^+^‐ATPase to provide the favourable Na electrochemical gradient. The Na^+^/K^+^‐ATPase, located in the BLM of crypt cells, was significantly stimulated in the crypt cells during chronic intestinal inflammation 27. However, this increase in Na^+^/K^+^‐ATPase activity during chronic intestinal inflammation was reversed to normal levels by ATK. The Na^+^/K^+^‐ATPase was not affected by ATK in crypt cells from the normal intestine (Fig. 1B; 6.0 ± 0.4 nmol/mg protein min in normal; 5.8 ± 0.2 in normal+ATK; 11.75 ± 0.5 in inflamed; 6.3 ± 0.2 in inflamed+ATK; *n* = 3, *P* < 0.01). Thus, at the cellular level, altered Na‐extruding capacity of crypt cells during chronic enteritis may, at least in part, contribute to the stimulation of NGcT.

### Effect of ATK treatment on NGcT in crypt cell BBMV

**Figure 2:**
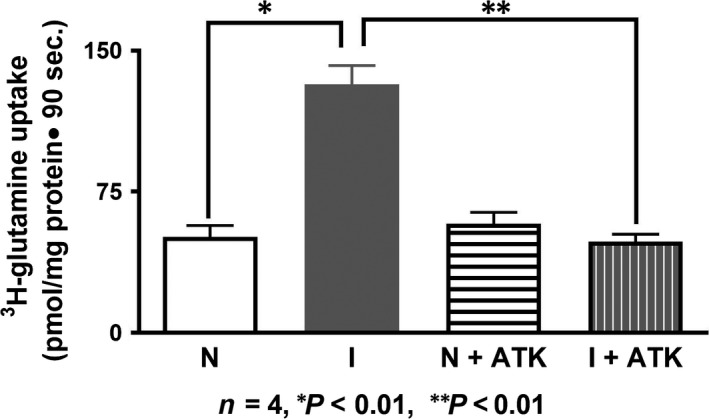
Effect of ATK on SN2 in crypt cells BBMV. Na‐dependent glutamine uptake was significantly increased in crypt cell BBMV from the chronically inflamed intestine. This inhibition was reversed with *in vivo* ATK treatment. NGcT was not affected by ATK treatment in the normal intestine.

To determine whether NGcT stimulation in crypt cells is mediated at the level of the cotransporter in the BBM, NGcT uptake was measured in crypt cell BBMV. NGcT was significantly stimulated in the crypt cell BBMV from the chronically inflamed intestine 27. However, when rabbits with chronic inflammation were treated with ATK, the stimulation of NGcT was abolished. In normal rabbits, ATK treatment had no effect on NGcT (Fig. 2; NGcT uptake in normal rabbits was 51 ± 6.0 pmol/mg protein 30 sec.; in chronically inflamed rabbits, it was 134 ± 5.8; in normal+ATK rabbits, it was 58 ± 6.0; and in inflamed+ATK rabbits, it was 48 ± 3.9; *n* = 3, *P* < 0.01). These data show that ATK treatment reverses the stimulation of NGcT at the level of the cotransporter in the BBM of crypt cells from the chronically inflamed intestine.

### Effect of ATK on NGcT kinetic parameters in crypt cells

**Figure 3:**
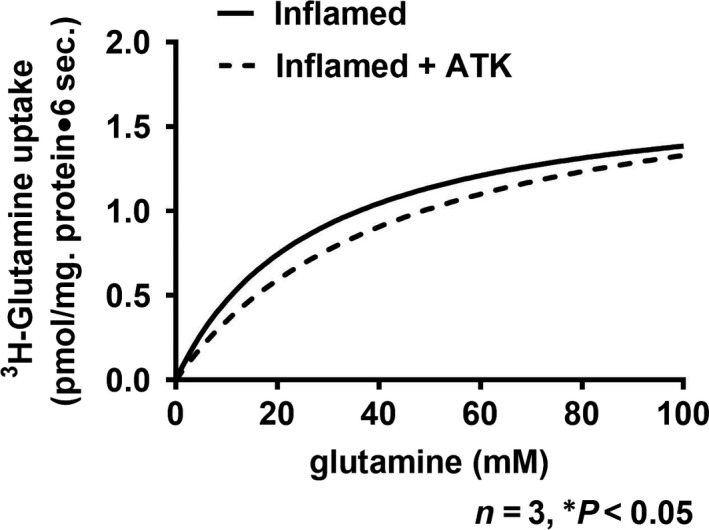
Kinetics of Na‐dependent glutamine uptake in villus BBMV. As the concentration of extravesicular glutamine was increased, the uptake of Na‐dependent glutamine also increased and subsequently became saturated in all conditions. The kinetic parameters derived from these data are shown in Table 1.

Kinetic studies were performed to determine the mechanism of the reversal of stimulation of NGcT. Na‐dependent glutamine uptake is shown as a function of varying concentrations of extravesicular glutamine at 6 sec. (Fig. 3). As the concentration of extravesicular glutamine was increased, glutamine uptake was stimulated and subsequently became saturated in all conditions. To derive the kinetic parameters, the data were analysed using GraphPad Prism 7 .

In the chronically inflamed intestine, NGcT is stimulated in crypt cells secondary to an increase in the affinity (1/*K*_m_) of the cotransporter for glutamine without a change in the number of cotransporters (*V*_max_). Treatment of rabbits with chronic enteritis with ATK reverses the stimulation by restoring the *K*_m_ without a change in the *V*_max_. (Table 1; *K*_m_ for Gln uptake in crypt cell BBMV was 51.8 ± 0.6 mM in the normal group, 18.1 ± 0.9 in inflamed group and 49.3 ± 3.6 in the inflamed+ATK group; *n* = 3, *P* < 0.05). These data indicated that the mechanism of reversal of SN2 stimulation by ATK was secondary to restoration of affinity of the cotransporter (*K*_m_) for glutamine without a change in the number of cotransporters (*V*_max_).

**Table 1:** Kinetic studies of Na‐glutamine cotransport in ATK‐treated crypt cell BBMV

	*V*_max_ (pmol/mg protein 6 sec.)	*K*_m_ (mM)
Control	2.2 ± 0.2	51.8 ± 0.6*
Inflamed	1.8 ± 0.1	18.1 ± 0.9*
Inflamed + ATK	2.1 ± 0.2	49.3 ± 3.6*

Kinetic parameters of Na‐glutamine cotransport in ATK‐treated crypt cell BBMV. In crypt cells, the maximal rate of uptake (*V*_max_) of NGcT was unchanged among the normal, inflamed and ATK‐treated groups. However, the affinity was significantly increased in chronic inflamed intestine, and this increased affinity was reversed by ATK treatment (*n* = 3, *P < 0.05).

### Quantitation of SN2 protein in crypt cell BBM in ATK treatment

**Figure 4:**
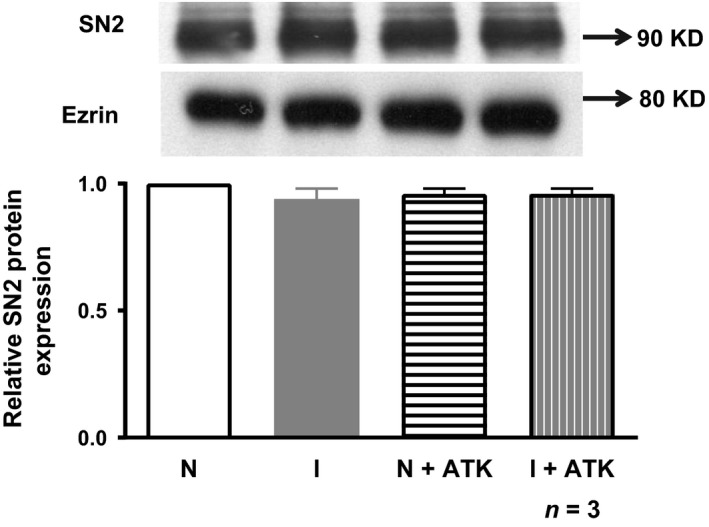
SN2 protein expression in crypt cell BBM. SN2 protein expression in crypt cell BBM from control, chronic enteritis and ATK‐treated rabbits. A representative Western blot of BBM SN2 protein is shown in the upper panel. In the lower panel, densitometric quantitation is shown. The relative protein expression levels of SN2 are unaffected among all the groups. Ezrin was used as internal control.

In the rabbit intestine, SN2 is the major NGcT in the BBM of crypt cells 2. Therefore, in the present study, immunoreactive protein levels of SN2 were determined in the crypt cell BBM for all experimental conditions. Analysis of the immunoblot for SN2 protein in the crypt cell BBM is shown in Figure 4. Densitometric quantitation of these blots demonstrated that the immunoreactive SN2 protein levels are unchanged in crypt cell BBM in normal, inflamed, normal+ATK or inflamed rabbits treated with ATK (Fig. 4). Thus, these studies are consistent with the kinetic studies above and indicate that the mechanism of stimulation of SN2 is secondary to enhanced affinity of the cotransporter for glutamine.

### NGcT in the crypt cells BBMV treated with PRX

**Figure 5:**
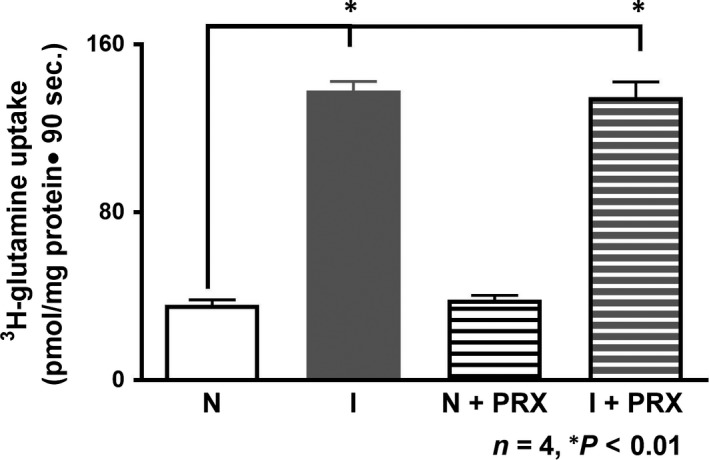
Effect of piroxicam on SN2 in crypt cells BBMV. Na‐dependent glutamine uptake was significantly increased in crypt cell BBMV from the chronically inflamed intestine. This stimulation was not reversed with *in vivo* piroxicam treatment. Na‐glutamine cotransport was not affected in the normal intestine treated with piroxicam. These data indicated that Na‐glutamine cotransport in crypt cells are likely not regulated by COX pathway metabolites in the chronically inflamed intestine.

NGcT in chronically inflamed crypt cells was significant stimulated 2. However, PRX treatment of the chronically inflamed rabbits failed to reverse this stimulation (Fig. 5). In normal rabbits, PRX had no effect on NGcT (Fig. 5; NGcT uptake in normal; 36 ± 2.3 pmol/mg protein 30 sec.; in inflamed: 138 ± 4.3; in normal+PRX: 38 ± 2.1; in inflamed+PRX; 135 ± 7.1; *n* = 3, *P* < 0.01). These results show that PRX failed to reverse the stimulation of NGcT uptake in crypt cells. Thus, these data demonstrate that during chronic intestinal inflammation, NGcT is not regulated by COX pathway metabolites.

### NGcT in the crypt cells BBMV treated with MK886

**Figure 6:**
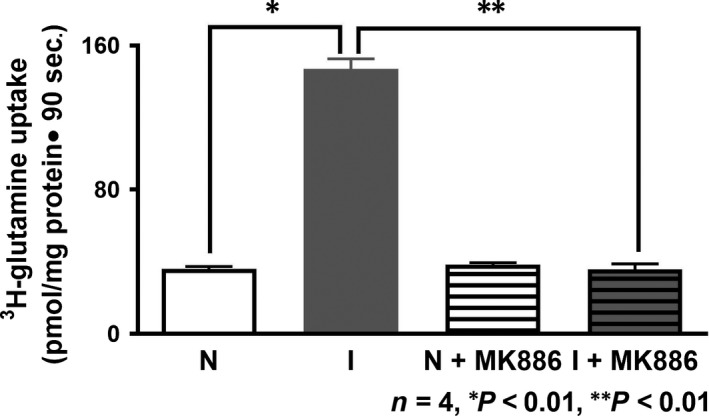
Effect of MK886 on SN2 in crypt cells BBMV. Na‐dependent glutamine uptake was significantly increased in crypt cell BBMV from the chronically inflamed intestine. This stimulation was reversed by *in vivo* MK886 treatment. Na‐glutamine cotransport was not affected in the normal intestine treated with MK886. These data indicate that Na‐glutamine cotransport in crypt cells likely regulated by LOX pathway metabolites in the chronically inflamed intestine.

In the BBMV of crypt cells, NGcT was significantly stimulated in the chronically inflamed intestine 27. This stimulation was abolished to near normal levels by MK886 treatment (Fig. 6). In normal rabbits, MK886 treatment did not have any effect on NGcT in crypt cell BBMV (NGcT uptake in normal: 36.2 ± 1.3 pmol/mg protein 30 sec.; in chronically inflamed, 147 ± 5.6; in normal+MK886, 38.4 ± 1.2; in inflamed+MK886, 35.8 ± 3.0; *n* = 3, *P* < 0.01). These data indicate that crypt cell BBMV NGcT in chronically inflamed intestinal crypt cells is likely regulated by the LOX pathway.

### Kinetic studies in crypts cells treated with MK886

**Figure 7:**
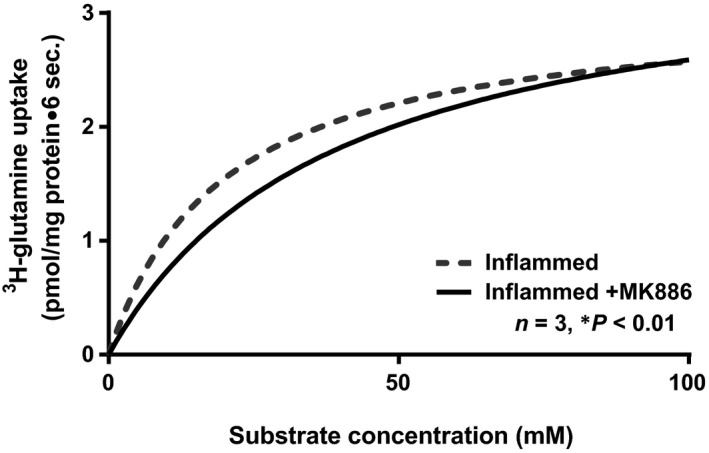
Kinetics of Na‐dependent glutamine uptake in crypt cell BBMV. As the concentration of extravesicular glutamine was increased, the uptake of Na‐dependent glutamine also increased and subsequently became saturated in all conditions. The kinetic parameters derived from these data are shown in Table 2.

To determine the mechanism of reversal of stimulation of NGcT by MK886 in the chronically inflamed intestine, kinetic studies were performed. NGcT uptake is shown as a function of varying concentrations of extravesicular glutamine at 6‐sec. (Fig. 7). Glutamine uptake was stimulated and subsequently became saturated in all conditions. To derive the kinetic parameters, the data were analysed using GraphPad Prism 7. In the chronically inflamed intestine, NGcT is stimulated in crypt cells secondary to an increase in the affinity (1/*K*_m_) of the cotransporter for glutamine (Table 2) without a change in the number of cotransporters (*V*_max_). Treatment with MK886 reversed the stimulation of NGcT in crypt cells by restoring the *K*_m_ without affecting the *V*_max_ (Table 2; *K*_m_ for glutamine uptake in crypt cell BBMV was 43.3 ± 0.06 mM in normal; 19 ± 0.57 in inflamed, and 43.9 ± 5.7 in inflamed+MK886; *n* = 3, *P* < 0.01). These data indicate that the mechanism of reversal of SN2 stimulation in the inflamed intestine by MK886 was secondary to restoration of affinity of cotransporter (*K*_m_) without a change in number of cotransporters.

**Table 2:** Kinetic studies of Na‐glutamine cotransport in MK886‐treated crypt cell BBMV

	*V*_max_ (pmol/mg protein 6 sec.)	*K*_m_ (mM)
Control	3.4 ± 0.32	43.3 ± 0.06*
Inflamed	3.0 ± 0.33	19 ± 0.57* ^,^**
Inflamed + MK886	3.9 ± 0.1	43.9 ± 5.7**

Kinetic parameters of Na‐glutamine cotransport in MK886‐treated crypt cell BBMV. In crypt cells, the maximal rate of uptake (*V*_max_) of Na‐glutamine cotransport was unchanged among the normal, inflamed and MK886‐treated groups. However, the affinity was significantly increased in the chronically inflamed intestine and this effect was reversed by MK886 treatment (*n* = 3, *P < 0.01, ** *P* < 0.001).

### Quantitation of SN2 protein in crypt cells BBMV in MK886 treatment

**Figure 8:**
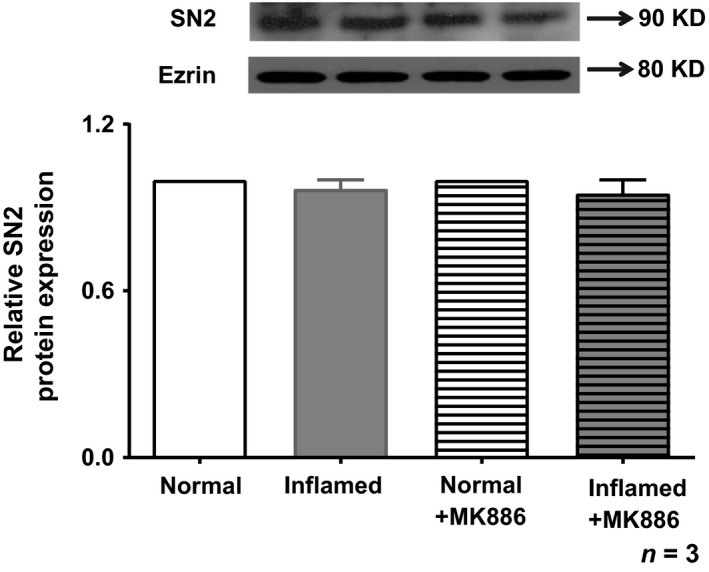
SN2 protein expression in crypt cell BBM. SN2 protein expression in crypt cell BBM from control, chronic enteritis and MK886‐treated rabbits. A representative Western blot of BBM SN2 protein is shown in the upper panel. Ezrin was used as loading control. In the lower panel, densitometric quantitation is shown. The relative protein expression levels of SN2 are unaffected among all the groups.

SN2 mediates NGcT in the BBM of intestinal crypt cells in rabbits 27. SN2 protein levels in crypt cell BBM were not altered significantly during chronic enteritis 27. Next, we determined the immunoreactive protein levels of SN2 in the crypt cell BBM in MK886‐treated rabbits. Analysis of the immunoblot for SN2 protein in the crypt cell BBM for all conditions is shown in Figure 8. SN2 protein levels are unaffected in crypt cell BBM from normal, inflamed, normal+MK886 or inflamed treated with MK886 (Fig. 8). Thus, in conjunction with kinetic studies, the mechanism of reversal of stimulation of SN2 in crypt cells during chronic intestinal inflammation by MK886 is secondary to restoration of the affinity for glutamine without a change in BBM cotransporter numbers.

**Figure 9:**
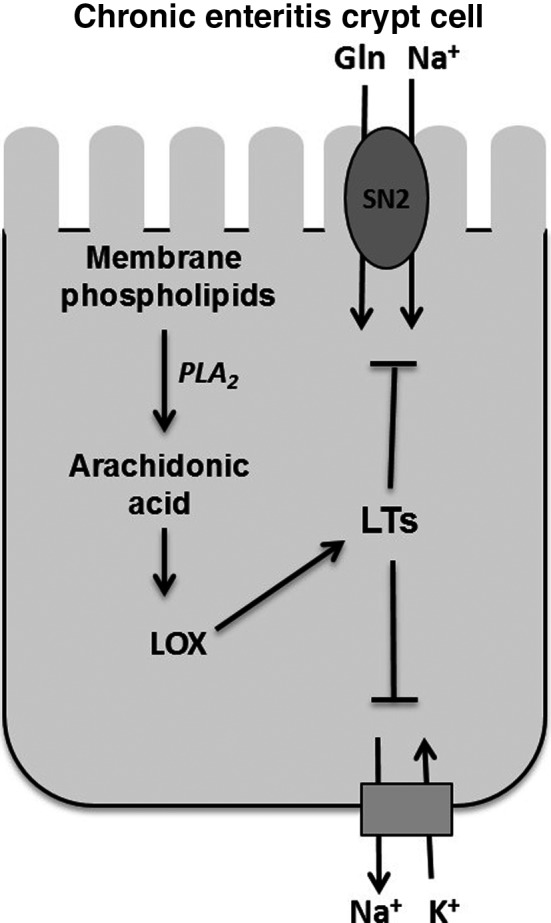
AA‐mediated regulation of NGcT. Na‐dependent glutamine cotransporter SN2 in crypt cells is facilitated by AA pathway particularly through LOX pathway during chronic intestinal inflammation.

## Discussion

Immune‐inflammatory mediators are known to regulate electrolyte absorption and secretion in the chronically inflamed intestine, and their role in the regulation of nutrient absorption during chronic enteritis is becoming more obvious. Alteration in glutamine assimilation in the chronically inflamed intestine has been reported 25, 27. However, the regulation of NGcT by AA pathway during chronic intestinal inflammation has not been reported. We have previously reported that NGcT is mediated by SN2 in rabbit intestinal crypt cells in the normal intestine 2 and that it is stimulated during chronic intestinal inflammation 27. In the present study, we show for the first time that SN2 in crypt cells during chronic intestinal inflammation is regulated by AA metabolites. Specifically metabolites of LOX, but not the COX pathway, regulate SN2 in crypt cells during chronic intestinal inflammation. Further research is needed to determine which metabolite of the LOX pathway is critical for this alteration in SN2 function. Once elucidated, targeting the LOX pathway with pharmacological inhibitors could prove beneficial in the clinic as a treatment option for IBD patients in the future.

In the rabbit model of chronic intestinal inflammation, we have shown that the functions of several solute transporters, both Na dependent and Na independent, are affected during chronic intestinal inflammation 5. In the present study, we found that SN2 activity was stimulated during the inflammatory state which was able to be reversed with MK886 and ATK treatments, but not PRX treatments. Our observations correlate with previous studies from our group which revealed that there was a decrease in the transporter activity of short‐chain fatty acid–bicarbonate exchanger 28, H‐dipeptide cotransporter 6, Na‐amino acid transporter 8, Na–glucose cotransporter (SGLT‐1) 9 and Na–bile acid transporter 10 during chronic intestinal inflammation. However, the mechanism of alteration of different cotransport processes appears to be unique in the chronically inflamed intestine. For example, the mechanism of inhibition of SGLT‐1 was secondary to a decrease in the *de novo* synthesis of SGLT‐1 in villus cells during chronic enteritis 9. In contrast, Na–neutral amino acid cotransporter (ASCT1) was inhibited during chronic intestinal inflammation secondary to a decrease in the affinity of the cotransporter for its substrate without a change in the number of cotransporters 29. Further, the distribution of nutrient transporters in villus and crypt cells is different. For example, it has been demonstrated that Na–glucose, Na–amino acid (alanine), Na–bile acid cotransporters and H‐dipeptide cotransporters are found on the brush border membrane (BBM) of the villus, but not in the crypt cells 4, 5, 6, 7, 8, 9, 10, 30. In fact, NGcT, specifically SN2, is the only nutrient absorptive process that has been found in crypt cells.

All Na‐nutrient cotransport processes in the intestine require the Na^+^/K^+^‐ATPase. This ubiquitous BLM transport protein is responsible for establishing and maintaining a high K(+) and low Na(+) concentration in the cytoplasm required for normal resting membrane potentials and critical for the functioning of Na‐dependent cotransporters 31, 32. Earlier studies have demonstrated that Na^+^/K^+^‐ATPase activity is altered during chronic intestinal inflammation 27. For example, in this study, at the cellular level, ATK‐mediated reversal of stimulation of SN2 in crypt cells was a result of restoration of Na^+^/K^+^‐ATPase activity in the BLM as well as the activity of cotransporters in the BBM. Finally, the differential mechanism of regulation of NGcT in crypt cells during chronic intestinal inflammation, specifically the altered affinity of SN2, suggests that a specific immune‐inflammatory mediator/pathway may be responsible for this alteration.

Similar to our observation in the present study, several researchers have previously shown arachidonic acid metabolite‐mediated inhibition of Na^+^/K^+^‐ATPase activity. In the rat cortical collecting duct, products of the COX pathway, specifically PGE2 was shown to inhibit the pump indirectly by decreasing intracellular Na 33. In another study, it was has been suggested that protein kinase A (PKA) or protein kinase C (PKC), a downstream signalling molecule of the COX/LOX pathway, affected Na^+^/K^+^‐ATPase through direct phosphorylation of the alpha subunit 34. In the present study, we show that the inhibition of phospholipase C by ATK treatment results in the restoration/reduction of Na/K‐ATPase to its normal levels, thus corroborating with the observations by other researchers. However, how and which COX/LOX pathway intermediates specifically regulate Na/K‐ATPase in crypt cells during chronic intestinal inflammation warrants future investigation.

The broad‐spectrum immunomodulators, like methylprednisolone 25 and ketotifen 35, reverse the stimulation of SN2 during chronic intestinal inflammation. However, these agents target upstream metabolites in the AA pathway at the level of PLA2. Therefore, their use complicates knowing which of the downstream pathways is predominantly responsible for the observed changes in SN2 activity. In this study, we used specific COX and LOX inhibitors, PRX and MK886, respectively, to delineate which pathway was the primary driving force in SN2 changes noted in chronic enteritis. LTs, formed by LOX, are well known pro‐inflammatory mediators and have been implicated in the pathophysiology of several inflammatory disorders, particularly asthma and IBDs 36, 37. LTD4 has been found to play a major role in the progression of intestinal inflammation 29. The involvement of the LOX pathway, but not COX, in the regulation of SN2 in crypt cells during chronic intestinal inflammation is consistent with previous studies that have shown that COX‐1 and COX‐2 expression is absent in crypt cells 38. Hence, the failure of PRX to reverse the stimulation of SN2/SNAT5 in crypt cells may be attributable to this. High levels of LTD4 have been detected in inflamed colonic mucosa from patients with ulcerative colitis 39. The effects of various LTs on chloride bicarbonate exchanger DRA and ion transport in the ileum and the colon have also been reported. Both LTD4 and LTE4 have been shown to alter intestinal absorptive and secretory pathways in some studies but not others 40, 41. Previously, our laboratory has shown that LTD4 alters the affinity of the Na–alanine cotransporter, ASCT1, secondary to changes in ASCT1 phosphorylation levels which are mediated by PKC 30. Further studies are needed to determine whether SN2 is regulated by similar mechanisms, such as activation of the PKC pathway by LTs present in the small intestine like LTD4.

In conclusion, the Na‐dependent glutamine cotransporter SN2 in crypt cells is stimulated in the small intestine during chronic inflammation secondary to enhanced affinity of the cotransporter for glutamine. The stimulation is not secondary to altered Na‐extruding capacity of the cells. Both inhibition of the release of AA and formation of LTs but not PGs reverse the stimulation of SN2 back to normal in crypt cells from the chronically inflamed intestine. Further, the mechanism of reversal of stimulation of SN2 by both ATK and MK886 was secondary to the restoration of affinity of the cotransporter (1/*K*_m_) without a change in the number of cotransporters. Thus, based on the above findings, it is likely that LOX, but not COX pathway, metabolites mediate the stimulation of SN2 in crypt cells during chronic intestinal inflammation.

## Conflict of interests

The authors have declared that no conflict of interests exist.
